# Therapeutic approaches for SAPHO syndrome from the perspective of pathogenesis: a review of the literature

**DOI:** 10.3389/fimmu.2025.1560398

**Published:** 2025-04-15

**Authors:** Yunuo Wang, Mengjiao Gu, Zixiang Zheng, Haixu Jiang, Luyao Han, Hanjing Huang, Yuanhao Wu, Chen Li

**Affiliations:** ^1^ First Teaching Hospital of Tianjin University of Traditional Chinese Medicine, National Clinical Research Center for Chinese Medicine Acupuncture and Moxibustion, Tianjin, China; ^2^ School of Chinese Materia, Beijing University of Chinese Medicine, Beijing, China; ^3^ Department of Dermatology, Tianjin Institute of Integrative Dermatology, Tianjin Academy of Traditional Chinese Medicine Affiliated Hospital, Tianjin, China; ^4^ Department of Rheumatology, Fangshan Hospital, Beijing University of Chinese Medicine, Beijing, China

**Keywords:** SAPHO syndrome, autoimmunity, infection, immune abnormality, bone metabolism, review

## Abstract

Synovitis, acne, pustulosis, hyperostosis and osteitis (SAPHO) syndrome is a rare autoinflammatory disease characterized by cutaneous manifestations and osteoarticular damage. The pathogenesis of SAPHO syndrome has not yet been elucidated, but studies have shown that the abnormal bone metabolism of patients with SAPHO syndrome is most likely due to localized infections that induce immune disorders in the body. Although no standardized treatment protocols exist, based on existing case studies and data from open studies, we propose that the treatment of SAPHO syndrome can be categorized into three areas according to the symptomatic manifestations of the disease: (1) control of focal infections using antibiotics and tonsillectomy; (2) administration of DMARDs to manage disease progression; and (3) bone remodeling therapy with bisphosphonates to address abnormal bone metabolism. Furthermore, a comprehensive treatment approach tailored to the clinical manifestations of the patient can effectively alleviate symptoms and enhance quality of life.

## 1 Introduction

Synovitis, acne, pustulosis, hyperostosis, and osteitis (SAPHO) syndrome, first described by rheumatologist Chamot in 1987, is a rare autoinflammatory disorder characterized by cutaneous manifestations and osteoarticular damage (1, 2). Currently, the incidence of SAPHO syndrome is 1 in 10,000 among Caucasians and 0.00144 in 10,000 among Japanese (3), and there is a paucity of large-scale data from China. SAPHO syndrome is more prevalent among middle-aged women (4), though cases have also been reported in children (5) and the elderly (6–9).

**Figure 1:**
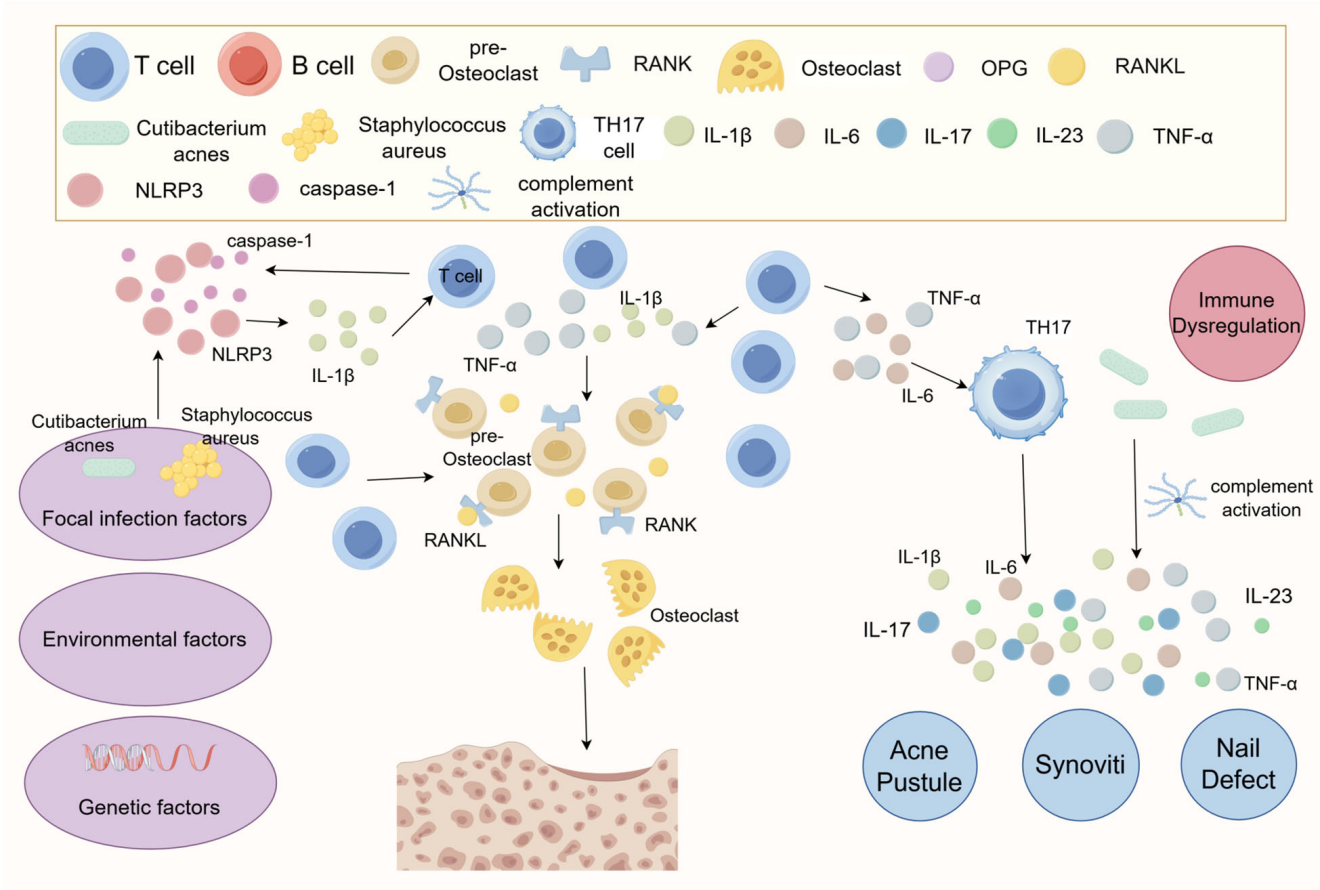
Diagram of the pathogenesis of SAPHO syndrome. The exact pathogenesis of SAPHO syndrome remains unclear, but it is suggested that it may involve an autoimmune response triggered by a low virulence pathogen. Abnormal bone metabolism in SAPHO syndrome patients is likely due to localized infections that induce immune disorders. Cutibacterium acnes is known to promote the production of IL-1β, which is associated with articular cartilage destruction, by activating NLRP3 inflammasomes and increasing caspase-1 activity. Additionally, Cutibacterium acnes can stimulate an immune response through complement activation and the production of cytokines such as IL-1, IL-8, and TNF-α. This immune response may lead to immune escape mechanisms, potentially related to abnormal T-cell activation, such as dysregulation in the balance between Treg and Th17 cells. This imbalance may further disrupt inflammatory cytokines, including IL-1β, IL-6, IL-17, IL-18, and TNF-α.

The pathogenesis of SAPHO syndrome remains unclear, with studies suggesting that it may result from an autoimmune response triggered by low-virulence pathogens (10). Various pathogens, including *Staphylococcus aureus* and *Cutibacterium acnes*, have been isolated from SAPHO patients by several researchers (11–22). Among these, *Cutibacterium acnes* promotes InterleukinIL-1β (IL-1β) production by activating NOD-Like Receptor Thermal Protein Domain Associated Protein 3 (NLRP3) inflammasomes and increasing caspase-1 activity (19, 20), a process linked to articular cartilage destruction (21). Additionally, *Cutibacterium acnes* induces an immune response by activating complement and promoting the production of cytokines, including IL-1, IL-8, and Tumor Necrosis Factor-α (TNF-α) (23). Palmoplantar Pustulosis (PPP), a common cutaneous manifestation of SAPHO, is also considered to be associated with chronic bacterial infections (24). Bacterial infections can abnormally activate the immune system, disrupting T-Helper 17 (Th17)/Regulatory T cells (Treg) cell homeostasis, promoting the expression of inflammatory factors, and activating osteoclasts. This cascade leads to bone destruction, osteomyelitis, and osteomalacia (25). Genetic factors may also contribute to the pathogenesis of SAPHO syndrome, with several cases of familial aggregation reported (26–28). Johannes Grosse and colleagues identified that chronic nonbacterial osteitis may be associated with Proline-Serine-Threonine Phosphatase Interacting Protein 2 (PSTPIP2) mutations; however, no pathogenic PSTPIP2 mutations have been found in human diseases (29). Thus, the pathogenesis of SAPHO syndrome may involve a complex interplay of infection, immunity, genetics, and bone metabolism disorders (**Figure 1**). Nevertheless, the exact pathogenesis of SAPHO syndrome remains elusive and necessitates further investigation.

Compared to other autoimmune diseases, SAPHO syndrome is a rare clinical autoimmune disease. Patients with SAPHO syndrome are often challenging to diagnose and are frequently misdiagnosed with PPP, leading to inadequate treatment. Additionally, most patients remain undiagnosed and untreated until they progress to more advanced skeletal manifestations. Treating SAPHO syndrome presents significant challenges, as clinicians must assess the efficacy of various treatments, weighing potential adverse effects to tailor an optimal regimen to the patient’s condition. This article discusses the recommendations for the use of medications in SAPHO syndrome more cautiously from the point of view of the pathogenesis of SAPHO syndrome (**Supplementary Table 1**, **Table 1**).

**Table 1:** Drug recommendation level.

Pharmacotherapy Disease domain			Minocycline	Tonsillectomy	Corticosteroids	csDMARDs		bDMARDs					tsDMARDs		Bisphosphonates			
						Methotrexate	Leflunomide	TNF-α inhibitor	IL-6 inhibitor	IL-17 inhibitor	IL-23 inhibitor	IL-1 inhibitor	JAKi inhibitor	Apremilast	Pamidronate Disodium Pentahydrate	Alendronate	Zoledronate	Ibandronate
osteoarticular	Osteitis	Synovitis (Peripheral joints and sternoclavicular joints)	/	+	+	+	+	++	+	++	+	+	++	/	+	/	++	/
		Anterior chest wall	+	+	+	+	+	++	-	++	+	-	+++	+	+++	+++	++	++
		Axial skeleton(Spineand Sacroiliac joints)	+	+	+	+	+	++	+	+	/	+	+++	+	+++	/	/	/
		Peripheral joints	+	+	+	+	+	+	-	++	+	-	+	+	++	/	/	/
		Mandible	+	/	+	+	/	+	+	/	/	/	+	/	++	/	++	/
		Long bone( Limbs)	/	+	/	+	/	+	-	/	/	/	+	+	++	+++	/	/
		Pelvis	/	/	/	+	/	+	/	+	/	/	/	/	++	/	/	/
Skin	PPP		+	+++	+	+	+	+	-	+	+	+	+	/	+++	+++	/	++
	Acne		+	-	+	+	/	++	-	+	+	/	+	/	+++	/	/	/
	Nail involvement		/	+	/	/	/	/	/	+	/	/	++	/	/	/	/	/

The color represents the type of study, + represents the level of our recommendation for the medication.

Red: Trial; Orange: open-label study; Blue: retrospective observational study; Green: Case series study; Gray: case report.

+: Recommended ++: Comparatively Recommended +++: Strongly Recommended -: Not Recommended /: Uncertain about the drug's efficacy or not mentioned in previous studies.

## 2 Treatment options for SAPHO syndrome

### 2.1 SAPHO syndrome and infection

Infection is an important factor in triggering autoinflammatory diseases (30). The two primary skin lesions of SAPHO syndrome, PPP and severe acne (SA), are strongly associated with infection. PPP is a chronic inflammatory skin condition characterized by recurrent aseptic pustules on the palms and soles and is linked to localized infections such as tonsillitis, dental infections, and sinusitis (31). Tonsillar epithelial cells in patients with PPP can secrete high levels of IL-6, leading to B cell stimulation and abnormal autoantibody production (32). In response to α-streptococcal antigen stimulation, tonsillar T cells in PPP patients express multiple receptors, such as Cutaneous Lymphocyte -Associated Antigen (CLA) and Chemokine C-C-Motif Receptor 6 (CCR6), which facilitate T cell migration into the skin and joints, exacerbating rashes and bone pain (33). In SAPHO syndrome, clinical manifestations of SA typically include polymerized and fulminant acne (34). Acne and pustulosis in SAPHO syndrome patients are strongly linked to infections (33). The isolation of various pathogens, including *Cutibacterium acnes*, has been reported in SAPHO syndrome patients (11–22). Infections caused by *P. acnes* promote activation of NLRP3 inflammasomes and IL-1β release, resulting in osteitis and enhanced differentiation of osteoblast mesenchymal stromal cells, a process linked to Forkhead Box O1 (FoxO1) deficiencies in osteoblasts (22). Rozin, A. P. et al. were the first to report successful treatment of a SAPHO syndrome patient using Co-Trimoxazole (CTM), an antibiotic with immunomodulatory properties, suggesting a potential link between *S. aureus* and SAPHO syndrome (35). Infection control strategies for SAPHO syndrome primarily involve antibiotic therapy and tonsillectomy.

#### 2.1.1 Antibiotics

Antibiotics are effective in the management of SAPHO syndrome (36). Tetracycline, clindamycin, minocycline, and azithromycin have been used to alleviate patient symptoms (4). Yasunobu Takizawa et al. reported a case of a 63-year-old female patient with SAPHO syndrome and severe co-infections who showed marked symptomatic improvement after treatment with minocycline alone (37). In an interventional study, Gunter Assmann et al. found that during a 4-month course of antibiotic therapy, the patient’s disease worsened after discontinuing the antibiotics, while controlling disease activity and improving dermatologic lesions and arthralgias during the treatment period (38). As clinical data on SAPHO syndrome continue to grow, more detailed antibiotic protocols are likely to emerge (4).

#### 2.1.2 Tonsillectomy

Tonsillitis may be a predisposing factor for SAPHO syndrome. Tonsils are considered a common site for chronic, subclinical infections (35). Previous studies have demonstrated significant efficacy of tonsillectomy in palmoplantar pustulosis (39), pustular arthritis (39), and immunoglobulin (Ig) A nephropathy (40–42). In a single-center retrospective study, investigators found that 67.2% of SAPHO syndrome patients had comorbid tonsillitis, and those with comorbid tonsillitis tended to present with more severe skin and nail lesions. Among patients treated with tonsillectomy, a significant improvement was observed in Visual Analogue Scale (VAS) and PalmoPlantar Pustulosis Area and Severity Index (PPPASI) scores after 3 months, suggesting that tonsillectomy may be associated with improvements in bone and skin symptoms in SAPHO syndrome patients (35). Although tonsillectomy is a relatively simple procedure, it carries certain risks, and the need for tonsillectomy should be carefully considered in the treatment of SAPHO syndrome.

### 2.2 SAPHO syndrome and immune abnormalities

Previous studies have shown that 49% of patients with SAPHO syndrome tested positive for *Acinetobacter* in bone biopsies (43) and that localized infections may be a causative factor in the immune disorders associated with SAPHO syndrome. However, an interventional study revealed that while disease activity was controlled during antibiotic use, patients experienced symptomatic deterioration after discontinuation of the drug (38). Therefore, autoimmune mechanisms may lead to immune escape during the progression of SAPHO syndrome. Its pathogenesis may be related to abnormal T-cell activation, such as an imbalance between Treg and Th17 cells, which in turn leads to the dysregulation of inflammatory cytokines including IL-1β, IL-6, IL-17, IL-18, and TNF-α (44). In clinical practice, Conventional Synthetic Disease-Modifying Antirheumatic Drugs (csDMARDs) are employed in the initial treatment of SAPHO syndrome and have shown good efficacy in managing peripheral joint symptoms and nail lesions (39). Corticosteroids are mainly used to relieve osteoarticular symptoms in patients with early SAPHO syndrome, although long-term use has been associated with a poor prognosis. Therefore, it is important to pay attention to the duration of treatment and to monitor the possibility of exacerbation of the symptoms involved in the use of corticosteroids. TNF-α levels are elevated in many inflammatory diseases of the skin and other organ systems, and its effects on adhesion molecule expression and immune activation establish it as a central mediator in many inflammatory skin diseases (45). Consequently, TNF-α inhibitors are often used as third-line agents following inadequate responses to Biological Disease-Modifying Anti-Rheumatic Drugs (bDMARDs) for rapid relief of inflammatory symptoms. IL-17, a signature cytokine secreted by Th17 cells, can synergize with TNF-α to exert pro-inflammatory effects, enhancing neutrophil migration to inflammatory sites (38). IL-17 inhibitors are therefore commonly used to improve cutaneous symptoms when TNF-α inhibitor therapy is ineffective (46). Additionally, IL-6 levels in SAPHO patients are significantly higher than those in healthy individuals, and the use of tocilizumab can alleviate the patients’ condition (40). Although some SAPHO patients exhibit relatively elevated levels of IL-1, there is limited evidence and few studies on its efficacy, so the future of IL-1-related treatments remains uncertain. Research into small molecule targeted drugs suggests that the Janus Kinase (JAK)/Signal Transducer and Activator of Transcription (STAT) signaling pathways play an important role in the pathogenesis of SAPHO syndrome. Among these, baricitinib, tofacitinib, and upadacitinib have been reported in treatment-related cases and have shown favorable therapeutic effects in improving skin and bone symptoms (41–45, 47).

#### 2.2.1 NSAIDs

Non-steroidal anti-inflammatory drugs (NSAIDs) are commonly regarded as the first-line pharmacological agents for pain relief and symptom control in SAPHO syndrome. They exhibit a rapid and pronounced effect on patients in the diagnostic phase of the disease (48), making them the top choice for treating patients with bone and joint symptoms (4). There is no clear superiority among different NSAIDs. To ascertain whether a patient is non-responsive to NSAIDs, it is advisable to administer full-dose medication for at least one month, ideally trying out two distinct NSAIDs (4). Nevertheless, relying solely on NSAIDs frequently fails to manage the condition, particularly for patients with extensive involvement. For instance, the efficacy of NSAIDs alone is limited in treating patients with extensive osteomyelitis (47). Furthermore, the gastrointestinal side effects associated with NSAIDs must be carefully considered during treatment (48).

#### 2.2.2 Corticosteroids

Corticosteroids are the mainstay of symptom control and treatment for many immune disorders (47). They may be prescribed to patients who are refractory to NSAIDs and have comorbid osteoarthritis (OA) (4).In a long-term follow-up study comprising 120 patients, corticosteroids were found to be an effective alternative therapy when nonsteroidal anti-inflammatory drugs (NSAIDs) failed to provide adequate symptom relief. Strikingly, patients treated with corticosteroids exhibited significant improvements in joint symptoms (47).Corticosteroids have a rapid onset but a short duration of action (48), yet there is limited research on their long-term efficacy in managing disease symptoms. Wang Lun et al. reported a case of SAPHO syndrome with elevated IgE levels, in which significant relief of clinical symptoms, as well as improvements in MRI and bone scan results, was observed following short-term corticosteroid application. This suggests that identifying disease-specific subtypes based on clinical presentation and IgE levels may help guide the use of corticosteroids in clinical practice (49). Furthermore, topical corticosteroids may be considered as a therapeutic option for managing dermatological manifestations in select clinical scenarios. A retrospective study has demonstrated that the combination of topical corticosteroids and PUVA therapy can also achieve satisfactory outcomes in skin improvement (50).However, other studies have found that topical corticosteroid injections primarily affect osteitis lesions (3) and that recurrence of skin and bone involvement occurs after tapering or discontinuation of the drug (51).Additionally, side effects such as osteoarthropathy and metabolic disorders associated with corticosteroid use have a serious impact on patient prognosis. Therefore, short-term, low-dose application of corticosteroids is recommended to minimize their potential side effects (49).

#### 2.2.3 csDMARDs: methotrexate and leflunomide

Although there is still no definitive diagnostic and therapeutic protocol for SAPHO syndrome, csDMARDs are frequently prioritized for treatment. The csDMARDs commonly used in the management of SAPHO syndrome are methotrexate and leflunomide. Clinical studies have shown that methotrexate can significantly improve joint inflammatory responses and is beneficial in managing SAPHO syndrome (52–56). As a traditional immunosuppressant, methotrexate inhibits T cell activity by promoting adenosine secretion from Treg cells, which in turn promotes Treg cell differentiation and suppresses the inflammatory response (56). Disease activity in SAPHO syndrome has been associated with an imbalance of Th17 and Treg cells (57), suggesting that methotrexate may be effective in improving the immune response in SAPHO patients. Leflunomide, employed for bone diseases, is also applicable in SAPHO syndrome treatment. Its mechanism of action is similar to that of methotrexate, as it inhibits T cell proliferation by reducing IL-2 production (58). Previous studies have confirmed the therapeutic efficacy of leflunomide in SAPHO syndrome patients (59, 60). However, recent investigations found that patients with SAPHO syndrome presenting with interstitial granulomatous dermatitis did not show symptom improvement after leflunomide treatment (61). In a cohort study, csDMARDs were found to be underutilized among children and adults with chronic nonbacterial osteomyelitis (CNO). Moreover, these agents demonstrated limited effectiveness in promoting disease remission (62). Therefore, it is important to consider the specific targets of csDMARDs in SAPHO syndrome treatment.

#### 2.2.4 bDMARDs

Previous studies have found that the pro-inflammatory phenotype of monocytes in SAPHO syndrome patients is associated with increased expression of IL-1β, IL-6, and TNF-α (63, 64). Moreover, Th17 cells are significantly elevated in patients with SAPHO syndrome compared to the healthy population (65). IL-6 is one of the major cytokines that induces polarization of Th17 cells, making targeting therapy against specific inflammatory factors a reasonable therapeutic strategy for SAPHO syndrome. Biological agents such as TNF-α inhibitors (infliximab, adalimumab, etanercept), IL-1 antagonists (anabolic leukocytoclasts), and IL-17 inhibitors (Secukinumab) have demonstrated good efficacy in treating SAPHO syndrome (66–74). However, uncertainties remain regarding the use of biological agents. Current safety data on the long-term use of biologics are primarily derived from studies on spondyloarthritis and psoriatic arthritis, with a notable lack of long-term safety assessments for their use in SAPHO syndrome (75, 76). Additionally, biologic therapy may be associated with side effects, such as the potential development of psoriatic dermatoses, increased risk of infections, or recurrence of palmar pustulosis during treatment (77, 78). Therefore, when choosing biologics to treat SAPHO syndrome, it is essential to consider the patient’s specific clinical symptoms to select the most appropriate treatment plan.

##### 2.2.4.1 TNF-α inhibitors

TNF-α is a pro-inflammatory cytokine, and anti-TNF-α therapeutic strategies play a significant role in the management of inflammatory diseases such as rheumatoid arthritis and inflammatory bowel disease (79). Currently, TNF-α inhibitors are clinically used as third-line drugs for SAPHO syndrome. Previous studies have shown that, for patients with inadequate response or failure to csDMARDs, TNF-α inhibitors such as Infliximab can more rapidly improve symptoms, including reducing pain, decreasing disease activity, significantly improving skin lesions, and decreasing bone destruction, providing a more sustained effect compared to csDMARD-based therapies (66–74, 80).In a single-center study involving 354 patients, individuals with involvement of the spine or sacroiliac joints exhibited significantly higher rates of TNF-α inhibitor use compared to those without such involvement. However, disease activity remained markedly elevated at baseline in these patients, suggesting that more refined management strategies may be required when axial joint involvement is present (81). Additionally, there may be side effects associated with TNF-α inhibitor therapy for SAPHO syndrome. Massara et al. found that two patients with SAPHO syndrome experienced other skin symptoms, including PPP recurrence, after the use of infliximab (5 mg/kg) (69). Similarly, Wagner et al. observed that patients developed bronchospasm after infliximab (5 mg/kg) treatment, which resolved upon switching to etanercept (67). Therefore, when using TNF-α inhibitors to treat SAPHO syndrome in clinical settings, careful consideration must be given to their safety profile and the potential for recurrence of skin symptoms.

##### 2.2.4.2 IL-6 inhibitors

Serum levels of IL-6 are significantly elevated in patients with SAPHO syndrome compared to the healthy population. As an inflammatory cytokine, IL-6 plays an important role in several inflammatory and autoimmune diseases (63). Tolizumab is a highly selective IL-6 inhibitor that can significantly reduce IL-6 levels and is well tolerated, although its efficacy in treating SAPHO syndrome remains limited. Sato et al. applied tozolizumab in the treatment of SAPHO syndrome and found that patients’ pain symptoms improved and MRI results showed relief of muscle high signal areas, but the symptoms of bone marrow edema did not improve (82). Furthermore, studies have indicated that patients with significantly elevated IL-6 levels experienced worsening bone and joint pain, skin herpes symptoms, and in two cases, reduced leukocyte levels, with these symptoms gradually disappearing after discontinuation of the drug (83, 84). Therefore, tolizumab is rarely considered as a therapeutic agent in the clinical treatment of SAPHO syndrome.

##### 2.2.4.3 IL-17 inhibitors

Firinu et al. found an increased number of Th17 cells in the peripheral blood of patients with SAPHO syndrome (65). Previous studies have shown that the activity of IL-17A is elevated in patients with PPP, and Th17 cells play a critical role in this inflammatory process (85–87). Therefore, the imbalance between Treg and Th17 cells is likely central to the development of skin symptoms in SAPHO syndrome. Current studies have found that IL-17 inhibitors such as Secukinumab and Brodalimumab are highly effective in ameliorating osteitis as well as severe skin symptoms in patients (46, 88–90). In addition, patients receiving TNF-α inhibitors have a 2.01-fold increased risk of inflammatory central nervous system disease compared to those not receiving TNF-α inhibitors (91). Interestingly, compared to TNF-α inhibitors, the IL-17 inhibitor Brodalimumab demonstrates superior efficacy in treating patients with comorbid Central Nervous System(CNS) disease, improving both peripheral joint symptoms and CNS complications (92). Therefore, IL-17 inhibitor therapy should be considered when csDMARDs and TNF-α inhibitors prove ineffective in managing skin symptoms or comorbid CNS symptoms. However, Secukinumab has been associated with colitis in some patients (65), and other patients have developed skin lesions following its use (61). Consequently, when using IL-17 inhibitors for SAPHO syndrome, physicians should advise patients to have regular follow-ups and promptly adjust the treatment strategy in the event of adverse effects.

##### 2.2.4.4 IL-23 inhibitors

The IL-23/IL-17 axis plays an important role in the pathogenesis of SAPHO syndrome (61). Therefore, compared to IL-17 inhibitors, IL-23 inhibitors are comparably effective in treating inflammatory diseases associated with SAPHO syndrome, particularly in improving skin symptoms. Currently, IL-23-related inhibitors that have been reported for the treatment of SAPHO syndrome include Ustekinumab, Tildrakizumab, and Risankizumab. In two reports on the use of Ustekinumab for treating SAPHO syndrome, researchers found that treatment-naïve patients who developed Interstitial Granulomatous Dermatitis (IGD) after csDMARDs treatment—with IGD potentially linked to leflunomide in one patient—experienced significant improvement in both arthritic symptoms and IGD after receiving Ustekinumab (61, 93, 94). In another case report, a patient with SAPHO syndrome and suppurative sweating adenitis, who had severe joint pain, was treated sequentially with antibiotics, isotretinoin, and adalimumab. This regimen provided relief of joint symptoms but only mild improvement in skin symptoms. However, the patient’s skin symptoms improved significantly after treatment with Risankizumab (94). Similarly, previous studies have shown that in patients with SAPHO syndrome refractory to both csDMARDs and adalimumab, joint and skin symptoms were completely eliminated following treatment with Tildrakizumab (95). Therefore, IL-23 inhibitors should be considered for SAPHO patients with refractory inflammatory cutaneous changes. There are currently no reports or studies addressing the adverse effects of IL-23 inhibitors; thus, while IL-23 inhibitors may serve as an alternative treatment option for patients with cutaneous involvement, potential adverse effects remain a concern.

##### 2.2.4.5 IL-1 inhibitors

There are still relatively few studies on IL-1 inhibitors for the treatment of SAPHO syndrome. A previous open study involving six patients with SAPHO syndrome treated with Anakinra therapy showed that the treatment alleviated joint and osteitis symptoms. IL-1 inhibitors demonstrated potential benefits for patients who had previously failed TNF-α inhibitors. However, two of these patients experienced local injection reactions and elevated transaminases, respectively (96). In a case series on chronic relapsing multifocal osteomyelitis, a child showed improvement in skin and joint symptoms following initial Anakinra therapy, but subsequently developed costochondritis and rash after 12 and 17 months of treatment, respectively (97). Therefore, the efficacy and safety of IL-1 inhibitors remain to be thoroughly investigated.

#### 2.2.5 Small molecule drugs

##### 2.2.5.1 JAK inhibitors

With the deepening understanding of autoimmune diseases, it has become clear that the JAK/STAT signaling pathway plays a crucial pathogenic role in these conditions, leading to an increased use of JAK inhibitors in clinically refractory Immune-Mediated Diseases (IMDs) (98, 99). Elevated serum levels of IL-17 and TNF-α in SAPHO patients are likely related to their pathogenesis, and JAK inhibitors target multiple inflammatory factors, such as IL-17 and TNF-α, thereby potentially influencing various downstream pathways involved in the treatment of SAPHO syndrome (44, 65, 100, 101). Some studies have demonstrated that JAK inhibitors are effective in treating SAPHO syndrome with extra-articular symptoms, suggesting a possible overlap in disease targets and superior performance in such cases (102–105).

Currently, the most commonly used JAK inhibitor in clinical practice is tofacitinib (102–110), although some patients may develop drug tolerance during treatment. Tofacitinib has shown efficacy in alleviating symptoms of PPP, osteitis, and acne associated with SAPHO syndrome, and is generally effective in treating comorbidities of SAPHO syndrome (102–105). However, a cohort study found that some patients experienced elevated Low-Density Lipoprotein (LDL) levels following tofacitinib treatment, which can contribute to cardiovascular events such as atherosclerosis (106). Therefore, it is crucial to monitor potential cardiovascular risks, particularly in elderly patients and those at high risk for cardiovascular events. Additionally, Shibata et al. reported a case of tofacitinib-induced PPP in a patient with juvenile idiopathic arthritis, which resolved upon discontinuation of the drug (111). This highlights the importance of close monitoring for potential cardiovascular risks and infections during treatment.

The use of baricitinib and upadacitinib in the treatment of SAPHO syndrome remains relatively limited. Previous studies on baricitinib have revealed individual variability in therapeutic efficacy, indicating it may not be suitable for all patients (112). Baricitinib has been shown to improve osteoarticular and cutaneous symptoms in SAPHO syndrome in two clinical studies (113, 114); however, additional evidence is needed to confirm its precise efficacy. We report a recent case of significant improvement in joint and skin symptoms in a SAPHO patient treated with upadacitinib (115). As a JAK1 inhibitor, upadacitinib has limited clinical applications reported to date and may represent a promising new therapeutic option.

##### 2.2.5.2 Apremilast

Apremilast (Otezla) is an oral small molecule phosphodiesterase 4 (PDE-4) inhibitor (116) that selectively targets PDE-4. It inhibits T cell activation by increasing intracellular cyclic Adenosine Monophosphate (cAMP) levels, which reduces the activities of pro-inflammatory cytokines such as IL-2, IL-8, Interferon-γ(IFN-γ), and TNF-α, thereby modulating the immune response in SAPHO (117). However, clinical evidence supporting Apremilast is currently limited, and its efficacy varies. Adamo et al. reported that Apremilast significantly alleviated skin and joint symptoms in patients (118). Moreover, studies indicate that Apremilast has a relatively favorable safety profile and advantages such as oral administration and no requirement for routine laboratory testing (117). However, Apremilast is associated with side effects including upper respiratory tract infections, nausea, and diarrhea (117). Additionally, Zhang et al. documented a case where Apremilast was ineffective as the initial treatment for SAPHO syndrome (119). In conclusion, as an emerging treatment, the clinical application of Apremilast requires further investigation to substantiate its efficacy and safety.

### 2.3 SAPHO syndrome and bone metabolism

Skeletal manifestations of SAPHO syndrome primarily involve osteitis and bone hypertrophy, which are due to an imbalance in bone remodeling between osteoclasts and osteoblasts. Receptor activator of nuclear factor-κB (RANK) is crucial for osteoclast differentiation and activation. In the presence of Macrophage Colony-Stimulating Factor (MCSF), Receptor Activator of Nuclear Factor-κ B Ligand(RANKL) binds to RANK on osteoclast precursors, promoting their differentiation into mature osteoclasts (120). Osteoprotegerin (OPG) inhibits osteoclast differentiation and bone destruction by binding to RANKL. The RANKL-RANK-OPG system is crucial in regulating both osteoclast differentiation and the immune response. Under physiological conditions, B cells secrete OPG, whereas in an inflammatory state, activated B cells secrete RANKL, which promotes osteoclast differentiation and accelerates bone resorption (121). T cells can promote RANKL secretion directly or indirectly through IL-7, and activated T cells also secrete osteoclastogenic factors that enhance osteoclast differentiation. Osteoclastogenic factors directly influence the secretion of IL-6, IL-10, Granulocyte-Macrophage Colony-Stimulating Factor (GM-CSF), and the JAK-STAT signaling pathway. These factors exacerbate the inflammatory response and promote osteoclast differentiation, leading to skeletal manifestations (122). Bisphosphonates inhibit osteoclast activity by binding specifically to hydroxyapatite on the bone surface, thereby preventing abnormal bone metabolism, resorption, and calcium mobilization. Furthermore, bisphosphonates possess anti-inflammatory properties by reducing pro-inflammatory cytokines such as IL-1, TNF-α, and IL-6, and by inhibiting the antigen-presenting capacity of macrophages (123, 124). Bisphosphonates are classified into three generations based on their development and into oral and intravenous formulations based on the route of administration. Previous studies indicate that the primary bisphosphonates used in the treatment of SAPHO syndrome include disodium pamidronate, alendronate sodium, zoledronate sodium, and ibandronate sodium.

#### 2.3.1 Pamidronate disodium

Disodium pamidronate, a second-generation bisphosphonate, is commonly administered intravenously for the treatment of SAPHO syndrome. Studies have demonstrated that disodium pamidronate significantly alleviates bone pain symptoms, typically administered in doses of 30 mg or 60 mg daily, though multiple infusions are often necessary (55, 125–130). Our findings indicate that disodium pamidronate not only significantly alleviated spinal bone marrow edema but also provided rapid pain relief and enhanced mobility (131). Guignard et al. reported that intravenous disodium pamidronate significantly decreased the urinary hydroxyproline-to-creatinine ratio, a marker of osteoclast activity and bone turnover (132). Additionally, disodium pamidronate has been shown to lower levels of inflammatory markers, reduce the need for other medications, and decrease relapse rates (132–134).

#### 2.3.2 Alendronate sodium

Alendronate sodium, a second-generation bisphosphonate, has been recommended for SAPHO syndrome treatment due to its convenient oral administration and favorable efficacy. Fioravanti et al. reported significant improvement in clinical symptoms and immunological indices in a SAPHO syndrome patient after 1 year of treatment with oral alendronate sodium (70 mg/week) (135). Ichikawa et al. described successful SAPHO syndrome treatment with oral alendronate sodium (5 mg/day) and concluded that oral administration is safer and more convenient than intravenous methods (136).

#### 2.3.3 Zoledronate sodium

Zoledronate sodium, a third-generation bisphosphonate, is commonly administered intravenously and has been found effective in treating SAPHO syndrome patients resistant to conventional therapies. Kopterides et al. treated a male patient with recurrent jaw swelling using zoledronate sodium, resulting in significant improvement or resolution of jaw pain and swelling after several injections, with corresponding improvements in bone scans (137). Just et al. described a female patient with persistent sternal pain who experienced sustained relief of pain symptoms, improved quality of life, and significant improvements in inflammatory markers and bone density following a single intravenous injection of zoledronate sodium (138). Our study demonstrated that zoledronate sodium significantly improved patients’ VAS, Bath Ankylosing Spondylitis Disease Activity Index (BASDAI), and Bath Ankylosing Spondylitis Functional Index (BASFI) scores (135). Additionally, our previous study indicated no significant difference in efficacy between zoledronate sodium and pamidronate sodium, but the incidence of adverse events was lower in the zoledronate sodium group. Moreover, zoledronate sodium has a shorter infusion time compared to pamidronate sodium, which can enhance patient compliance (135).

#### 2.3.4 Ibandronate sodium

Ibandronate sodium, a third-generation bisphosphonate, is commonly administered intravenously for treating SAPHO syndrome. Soyfoo et al. reported a case involving a 22-year-old male patient with SAPHO syndrome who received zoledronate sodium 5 mg/month for recurrent anterior chest wall pain (139). This treatment provided rapid symptomatic relief but had a limited duration of action. In the second year, the patient was treated with ibandronate sodium 3 mg/month for recurrent symptoms, which provided both rapid and long-lasting relief and improved radiological findings. Gil et al. described a 50-year-old female patient with poor response to NSAIDs who experienced significant relief from joint pain and normalization of inflammatory markers following the administration of ibandronate sodium 150 mg/month and prednisolone 5 mg/day, though skin symptoms showed no significant improvement (140). Few reports exist on the use of ibandronate sodium for SAPHO syndrome, and its efficacy remains to be fully established.

#### 2.3.5 Technetium Methylene Diphosphonate

Technetium Methylenediphosphonate (Tc-MDP) is currently used for radionuclide scanning, early diagnosis of malignant bone tumors and their metastases, and clinical diagnosis of osteitis and metabolic bone diseases. Bone scanning with ^99m^Tc-MDP can be used to detect osteitis in patients with SAPHO syndrome. ^99m^Tc-MDP, a novel bisphosphonate, has demonstrated greater efficacy in bone protection compared to traditional immunosuppressants in other rheumatological and immunological diseases (141). Shao et al. treated a SAPHO syndrome patient with severe chronic mandibular osteomyelitis using ^99m^Tc-MDP, resulting in significant improvement in both systemic symptoms and mandibular bone damage (142). Despite the significant clinical efficacy of ^99m^Tc-MDP and bisphosphonates in treating bone resorption disorders such as SAPHO syndrome, the risk of Bisphosphonate-Related Osteonecrosis of the Jaw (BRONJ) as a potential complication must be considered (143).

## 3 Other considerations

The skin symptoms of SAPHO syndrome typically manifest as severe acne, nail changes, and PPP, which not only affect patients’ appearance but may also lead to significant psychological and social dysfunction. From the perspective of pathogenesis, the skin manifestations mentioned above are often triggered by underlying inflammation. Based on our single-center experience, we propose that minocycline can be effective for the treatment of acne. For pustular psoriasis, nail involvement, and other skin symptoms, we recommend the use of JAK inhibitors. In addition, topical skin treatments can also be considered as an option. We reviewed the relevant literature and summarized the therapeutic strategies for topical treatments in patients with SAPHO syndrome. Investigators have commonly used retinoids and adapalene for the treatment of mild to moderate acne, or clindamycin and erythromycin antibiotics to improve inflammatory acne (144). Moreover, a study found that the combination of corticosteroids and PUVA therapy also demonstrated good efficacy in improving skin symptoms (50).Therefore, topical skin treatments should also be given attention for symptom improvement.

## 4 Conclusion

**Figure 2:**
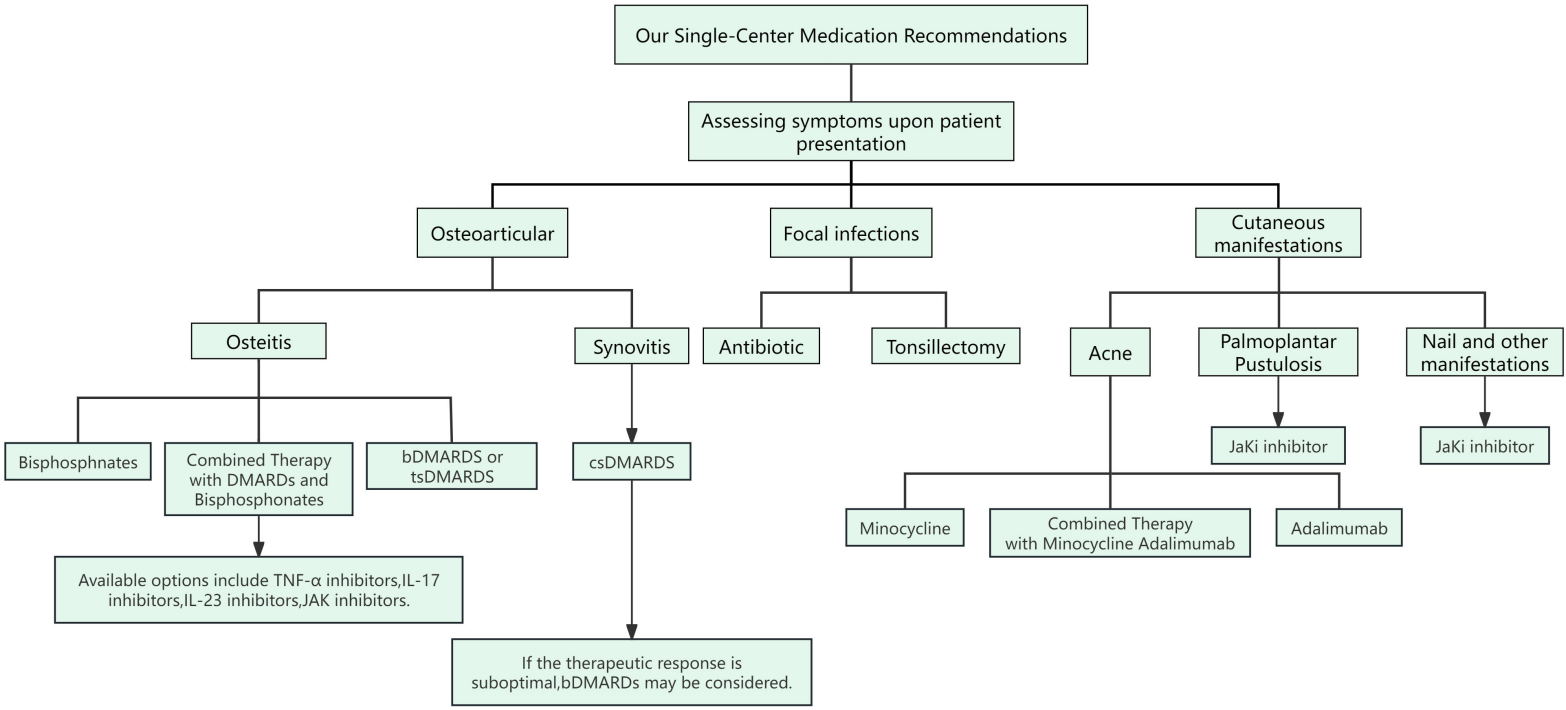
Our Single-Center Medication Recommendations for SAPHO Syndrome. Clinicians need to assess the patient’s symptoms to determine the appropriate medication. Patients with SAPHO syndrome present with three main types of symptoms: osteoarticular manifestations, localized infections, and skin involvement. Osteoarticular Involvement: For synovitis, conventional synthetic disease-modifying antirheumatic drugs (csDMARDs) are used. In cases of osteitis, biological DMARDs (bDMARDs) and bisphosphonates are recommended. If these treatments are unsatisfactory, additional bDMARDs such as TNF-α inhibitors, IL-17 inhibitors, IL-23 inhibitors, and JAK inhibitors are suggested. Localized Infections: For symptoms of focal infection, treatment options include antibiotics or tonsillectomy. Skin Involvement: Skin manifestations in SAPHO syndrome often include acne, pustular psoriasis (PPP), and nail changes. Minocycline may be used for acne. For PPP, nail involvement, and other skin symptoms, JAK inhibitors are recommended.

SAPHO syndrome is a rare autoinflammatory disease characterized by cutaneous manifestations and osteoarticular damage. The primary goal of treatment is to alleviate joint symptoms and skin lesions while preventing recurrence of the condition. We propose a combined and stratified treatment approach targeting the multifaceted pathogenesis of SAPHO syndrome. This strategy aims to achieve comprehensive disease control by eliminating focal infections, correcting immune dysregulation, and improving abnormal bone metabolism. Specifically, the treatment plan includes: (i) using antibiotics and tonsillectomy to control focal infections; (ii) employing DMARDs to manage disease progression; and (iii) using bisphosphonates to treat abnormal bone metabolism and promote bone remodeling. However, despite the success of these therapeutic approaches in some cases, the treatment of SAPHO syndrome still faces many challenges, including the lack of unified diagnostic criteria and treatment strategies. Overall, treating SAPHO syndrome necessitates an individualized plan tailored to each patient, with regular monitoring of efficacy and side effects to adjust the treatment regimen as needed (**Figure 2**).

## Glossary

SAPHO: Synovitis, Acne, Pustulosis, Hyperostosis, and Osteitis
DMARDs: Disease-Modifying Anti-Rheumatic Drugs
IL-1β: Interleukin-1β
NLRP3: NOD-Like Receptor Thermal Protein Domain Associated Protein 3
TNF-α: Tumor Necrosis Factor-α
PPP: Palmoplantar Pustulosis
Th17: T-Helper 17
Treg: Regulatory T Cells
PSTPIP2: Proline-Serine-Threonine Phosphatase Interacting Protein 2
SA: Severe Acne
CLA: Cutaneous Lymphocyte-Associated Antigen
CCR6: Chemokine C-C Motif Receptor 6
FoxO1: Forkhead Box O1
CTM: Co-Trimoxazole
Ig: Immunoglobulin
VAS: Visual Analogue Scale
PPPASI: Palmoplantar Pustulosis Area and Severity Index
csDMARDs: Conventional Synthetic Disease-Modifying Anti-Rheumatic Drugs
bDMARDs: Biological Disease-Modifying Anti-Rheumatic Drugs
JAK/STAT: Janus Kinase/Signal Transducer and Activator of Transcription
NSAIDs: Nonsteroidal Anti-Inflammatory Drugs
OA: Osteoarthritis
CNS: Central Nervous System
IGD: Interstitial Granulomatous Dermatitis
IMDs: Immune-Mediated Diseases
LDL: Low-Density Lipoprotein
PDE-4: Phosphodiesterase 4
cAMP: Cyclic Adenosine Monophosphate
IFN-γ: Interferon-γ
RANK: Receptor Activator of Nuclear Factor-κB
MCSF: Macrophage Colony-Stimulating Factor
RANKL: Receptor Activator of Nuclear Factor-κB Ligand
OPG: Osteoprotegerin
GM-CSF: Granulocyte-Macrophage Colony-Stimulating Factor
BASDAI: Bath Ankylosing Spondylitis Disease Activity Index
Tc-MDP: Technetium Methylene Diphosphonate
BRONJ: Bisphosphonate-Related Osteonecrosis of the Jaw
BASFI: Bath Ankylosing Spondylitis Functional Index
