# Supplementary material for: Therapeutic approaches for SAPHO syndrome from the perspective of pathogenesis: a review of the literature
Source: Front Immunol. 2025 Apr 15;16:1560398. doi: 10.3389/fimmu.2025.1560398 (PMC12037609; doi:10.3389/fimmu.2025.1560398)
Supplement: Supplementary file 1 [file Table1.docx]

Supplementary Table1:Summary of recent Pharmacotherapeutic Approaches of SAPHO Syndrome

| **Categories** |  | **Drugs** | **Publication information** | **Study taype** | **Subject** | **Country** | **Treatment duration** | **Outcome** | **Adverse events** |
| --- | --- | --- | --- | --- | --- | --- | --- | --- | --- |
| corticosteroid |  | methylprednisolone | Wang, Lun et al，2019^[1]^ | case report | 1 | China | 5 weeks | Significant improvement in PPP and nail lesions was observed. | NA |
| tonsillectomy |  | tonsillectomy | Xiang, Yirong et al，2021^[2]^ | A retrospective observational study | 58 | China | Gather clinical data on bone pain, skin, and nail lesion severity. Refer patients with tonsil issues (hypertrophy, congestion, secretion, stones) to ENT for evaluation. Follow up with the 7 post-tonsillectomy patients. | Tonsillectomy may improve bone and skin symptoms in SAPHO syndrome patients. | NA |
| minocycline |  | minocycline | Takizawa, Yasunobu et al,2014^[3]^ | case report | 1 | Japan | NA | The patient showed a favorable condition. | NA |
| csDMARDs |  | methotrexate | Freira S et al，2014^[4]^ | case report | 1 | Portugal | NA | Bone scan also improved remarkably. | NA |
|  |  |  | Akçaboy M et al，2017^[5]^ | case report | 1 | Turkey | 8 months | Bone scan also improved remarkably. | NA |
|  |  |  | Azevedo VF et al，2011^[6]^ | case report | 1 | Portugal | NA | Symptoms clinically stable. | NA |
|  |  |  | Aljuhani F et al，2015^[7]^ | Retrospective single center study | 4 | France | NA | 2 patients and was ineffective, partially effective in 1 | NA |
|  |  |  | Hayem G et al，1999^[8]^ | long-term follow-up study | 10 | France | 5 years | Results were good in 4 cases, partially satisfactory in 1, disappointing in 2, and unknown in 3. | NA |
|  |  | leflunomide | Cornillier H et al，2016^[9]^ | case report | 1 | France | 1 month | Papules located on the limbs and the face . | Papules and IGD |
|  | TNF-α inhibitors | Infliximab | Olivieri et al，2002^[10]^ | Case series | 2 | Italy | 6 weeks | Case 1.the disease has remained in remission so far. Case 2.osteitis disappeared | NA |
|  |  |  | Iqbal M et al，2005^[11]^ | Case report | 1 | USA | 11 months | The synovitis,osteitis completely resolved. | NA |
|  |  |  | Massara A et al，2006^[12]^ | Case series | 4 | Italy | 8 months | Case 1.osteitis decreased dramatically. Case 2.osteoarticular symptoms completely resolved. Case 3. osteoarticular remission persists. Case 4. complete resolution of osteitis | Case 1.papulopustular involving  Case 2.pustular lesions appeared |
|  |  |  | Sabugo F et al，2008^[13]^ | Case series | 1 | Chile | 6 weeks | synovitis disappeared and pustules decreased. | Exhibit symptoms of hyperthyroidism |
|  |  | Adalimumab | Garcovich S et al，2012^[14]^ | Case report | 1 | Italy | 24 months | Complete remission of clinical symptoms | NA |
|  |  |  | Castellví I et al，2010^[15]^ | Case report | 1 | Spain | 4 weeks | Has not yet referred any osteitis . | NA |
|  |  | Entercept | Sáez-Martín LC et al，2015^[16]^ | Case report | 1 | Spain | 12 weeks | The articular involvement showed a favorable progress. | NA |
|  |  |  | Zhang LL et al，2012^[17]^ | Case report | 1 | China | 4 months | Joint symptoms improved notebly. | NA |
|  |  |  | Wagner AD et al，2002^[18]^ | Case series | 2 | USA | 7 months | Case 1.MRI demonstrated an improvement of synovitis. Case 2.systemic inflammation decreased. | Case 2.brief episodes of shortness of breath 3 hours after each infusion |
|  |  | Tocilizumab | Sato H et al，2017^[19]^ | Case series | 2 | Japan | Case 1.4 weeks Case 2.10 months | Case 1.osteitis improved. Case 2.bilateral lower leg pain improved. | NA |
|  |  |  | Fujita S et al，2015^[20]^ | Case report | 1 | Japan | 3 weeks | The treatment normalized CRP levels and resolved osteitis . | presented with intractable  anterior chest pain and a swollen and  tender anterior sternum surface. |
|  |  |  | Sun XC et al，2018^[21]^ | Case series | 2 | China | Case 1.2 days Case 2.1 month | Case 1.osteoarticular and cutaneous manifestations became aggravated.  Case 2. osteitis rapidly resolved. | Case 1.transient severe neutropenia ,experienced osteitis, pustular rashes on the upper and lower limbs. |
|  | IL-17 inhibitors | Secukinumab | Ji Q et al，2022^[22]^ | Case report | 1 | China | 4 months | Complete remission of skin symptom. | NA |
|  |  |  | Wang L et al，2021^[23]^ | Case series | 4 | China | 24 weeks | Case 1. PPP improved, VAS50 & BASDAI50 & ASDAS-MI achieved.  Case 2. PPP & PV improved, nail lesions stable, BASDAI50 & ASDAS-MI achieved, VAS50 not achieved.  Case 3. PPP & PV improved, nail lesions improved, VAS50 & BASDAI50 & ASDAS-MI achieved.  Case 4. PPP improved, PV improved with IL-17 inhibitor, nail lesions improved, VAS50 & BASDAI50 & ASDAS-MI achieved. | NA |
|  |  |  | Funabiki M et al，2023^[24]^ | Case report | 1 | Japan | 50 weeks | Clinical symptoms including  the severe neck pain were resolved immediately. | NA |
|  |  | Brodalumab | Leloup, P et al，2013^[25]^ | Case report | 1 | France | 3 months | The joint pain improved. | NA |
|  | IL-23 inhibitors | Ustekinumab | Flora A et al，2021^[26]^ | Case report | 1 | Australia | 9 months | Complete resolution of symptoms . | NA |
|  |  | Risankizumab | Licata G et al，2021^[27]^ | Case report | 1 | Italy | at weeks 0, 4, and  every 12 weeks | Great clinical response with complete  elimination of joint pain and cutane- ous symptoms | NA |
|  |  | Tildrakizumab | Wendling D et al，2012^[28]^ | open study | 6 | France | once | Five patients experienced improvement in their symptoms. | Injection site reaction; |
|  | IL-1 inhibitor | Anakinra | Eleftheriou D et al，2010^[29]^ | Case series | 1 | British | 12 months | All symptoms resolved. | costochondritis and psoriasis-like rash |
|  |  |  | Yang Q et al，2018^[30]^ | Case report | 1 | China | 12 weeks | Improvement of symptoms. | NA |
| small molecule drugs | JAKi inhibitors | Tofacitinib | Cao F et al，2023^[31]^ | Case report | 1 | China | NA | Acne, swelling and pain were effectively alleviated. | NA |
|  |  |  | Yuan F et al，2022^[32]^ | Case report | 1 | China | 1 month | Rash subsided significantly,and the chest and back pain osteitis relieved. | NA |
|  |  |  | Ru C et al，2023^[33]^ | Case report | 1 | China | 6 months | Skin symptoms gradually relieved, and osteitis significantly reduced. | NA |
|  |  |  | Liu Y et al，2023^[34]^ | Case report | 1 | China | 3 months | The swelling and pain of left wrist disappeared | NA |
|  |  |  | Wang R et al，2023^[35]^ | Case report | 1 | China | 2 months | The ulcer was completely healed . | NA |
|  |  |  | Baisya R et al，2023^[36]^ | Case report | 1 | India | 3 months | Resolutions of symptoms achieved. | NA |
|  |  |  | Yang J et al，2024^[37]^ | Case report | 1 | China | 3 months | The skin lesions disappeared , and osteoarticular pain were significantly relieved. | NA |
|  |  |  | Liu S et al，2023^[38]^ | case series | 5 | China | 12 weeks | 5 patients’ clinical scores and laboratory indexes decreased in different degrees. | NA |
|  |  | Baricitinib | Ma M et al，2023^[39]^ | Case report | 1 | China | 3 months | Bone pain and pustules were all relieved. | NA |
|  |  | Upadacitinib | Adamo, S et al，2018^[40]^ | Case report | 1 | Switzerland | 6 months | Itching and skin pustules are alleviate  Itching and skin pustules are alleviated | Headaches, diarrhea, nausea, and vomiting were side effects from apremilast, which resolved spontaneously. |
|  |  | Apremilast | C. Li et al，2019^[41]^ | Prospective clinical trial | 30 | China | 3 days, at baseline and 3 months later. | Inflammation and osteitis improved | NA |
| Bisphosphonates |  | Pamidronate disodium | H. Amital et al，2004^[42]^ | open-label study | 10 | Israel | Patients got pamidronate IV in 1 hour. No response: next dose in 1 month; partial: another in 4 months | Joint and skin symptoms improved | NA |
|  |  |  | C. Kerrison et al，2004^[43]^ | A retrospective observational study | 7 | Britain | 30 days | Osteitis improved | NA |
|  |  |  | Valls-Roc, M., et al，2005^[44]^ | Case series study | 6 | Germany | On days 1, 7, 14, 28 and 56 | Osteitis improved | 1 patient had fever; 2 had superficial phlebitis. |
|  |  |  | E. Solau-Gervais et al，2006^[45]^ | open-label study | 13 | France | Over 6 months | Osteitis and skin symptoms improved | NA |
|  |  |  | M. Colina et al，2009^[46]^ | Prospective clinical trial | 14 | Italy | 3 days | 12 patients showed improvement of synovitis, osteitis, acne and hand and foot pustules, respectively | NA |
|  |  |  | Emilie Delattre et al，2014^[47]^ | A retrospective observational study | 22 | France | Every 4 weeks if needed. | Effective in 13 patients, partially effective in 8 patients, and completely ineffective in 1 . | 11 patients: flu-like (5), fever (3), low calcium, pink eye, headache, vein inflammation. |
|  |  |  | P. A. COURTNEY et al，2002^[48]^ | case report | 1 | Britain | Every 3 months, for 7 times in a row. | The osteitis improved significantly. | NA |
|  |  |  | H. MARSHALL et al，2002^[49]^ | case report | 1 | Britain | Over 5 months | The osteitis was relieved and the skin pustules were not improved. | NA |
|  |  | Alendronate  sodium | S. Guignard et al，2002^[50]^ | A retrospective observational study | 5 | France | 3 days | Four patients had reduced pain, one patient had a longer interval . | NA |
|  |  |  | Nan Wu et al,2020^[51]^ | A single cohort, open-label study | 25 | China | IV for 3 days, at start and 3 months later | A total of 13 and 11 patients responded to the first and second treatments. | Fever (71.5%), low calcium (30.0%), mild GI upset (22.0%). |
|  |  |  | Faisal Aljuhani et al,2015^[7]^ | A retrospective observational study | 26 | France | 3days | 18 patients experienced reduction in pain | 7 patients developed influenza syndrome. |
|  |  |  | Jiro Ichikawa et al，2009^[52]^ | case report | 1 | Japan | NA | Painful joints return to normal. | NA |
|  |  |  | S. Liu et al，2023^[53]^ | A retrospective observational study | 30 | China | 3 days | VAS,BASDI, and BASFI scores significantly improved. | NA |
|  |  | Zoledronic  sodium | Petros Kopterides et al，2004^[54]^ | open-label study | 1 | Greece | 18 months | The pain disappeared, and bone scans showed significant improvement | NA |
|  |  | Ibandronate  sodium | Alexander Just et al，2008^[55]^ | case report | 1 | Germany | 3 days | The patient reported significantly improved quality of life and continued regression of pain symptoms. | NA |
|  |  |  | Muhammad S. Soyfoo et al，2010^[56]^ | case report | 1 | Belgium | 3 months | Dramatic improvement of osteitis. | NA |

**References:**

[1]. Wang, L., et al., Long-term remarkable remission of SAPHO syndrome in response to short-term systemic corticosteroids treatment in an immunoglobulin E elevated patient: A case report. Medicine (Baltimore), 2019. 98(27): p. e16045.

[2]. Xiang, Y., et al., Tonsillitis as a possible predisposition to synovitis, acne, pustulosis, hyperostosis and osteitis (SAPHO) syndrome. Int J Rheum Dis, 2021. 24(4): p. 519-525.

[3]. Takizawa, Y., et al., Severe inflammation associated with synovitis, acne, pustulosis, hyperostosis, osteitis (SAPHO) syndrome was markedly ameliorated by single use of minocycline. Mod Rheumatol, 2014. 24(6): p. 1015-8.

[4]. Freira, S., et al., SAPHO syndrome in an adolescent: a clinical case with unusual severe systemic impact. J Adolesc Health, 2014. 55(2): p. 304-6.

[5]. Akçaboy, M., et al., Successful treatment of a childhood synovitis, acne, pustulosis, hyperostosis and osteitis (SAPHO) syndrome with subcutaneous methotrexate: A case report. Turk J Pediatr, 2017. 59(2): p. 184-188.

[6]. Azevedo, V.F., et al., [Methotrexate to treat SAPHO syndrome with keloidal scars]. Acta Reumatol Port, 2011. 36(2): p. 167-70.

[7]. Aljuhani, F., et al., The SAPHO syndrome: a single-center study of 41 adult patients. J Rheumatol, 2015. 42(2): p. 329-34.

[8]. Hayem, G., et al., SAPHO syndrome: a long-term follow-up study of 120 cases. Semin Arthritis Rheum, 1999. 29(3): p. 159-71.

[9]. Cornillier, H., et al., Interstitial granulomatous dermatitis occurring in a patient with SAPHO syndrome one month after starting leflunomide, and subsequently disappearing with ustekinumab. Eur J Dermatol, 2016. 26(6): p. 614-615.

[10]. Olivieri, I., et al., Successful treatment of SAPHO syndrome with infliximab: report of two cases. Ann Rheum Dis, 2002. 61(4): p. 375-6.

[11]. Iqbal, M. and M.S. Kolodney, Acne fulminans with synovitis-acne-pustulosis-hyperostosis-osteitis (SAPHO) syndrome treated with infliximab. J Am Acad Dermatol, 2005. 52(5 Suppl 1): p. S118-20.

[12]. Massara, A., P.L. Cavazzini and F. Trotta, In SAPHO syndrome anti-TNF-alpha therapy may induce persistent amelioration of osteoarticular complaints, but may exacerbate cutaneous manifestations. Rheumatology (Oxford), 2006. 45(6): p. 730-3.

[13]. Sabugo, F., et al., Infliximab can induce a prolonged clinical remission and a decrease in thyroid hormonal requirements in a patient with SAPHO syndrome and hypothyroidism. Clin Rheumatol, 2008. 27(4): p. 533-5.

[14]. Garcovich, S., et al., Long-term treatment of severe SAPHO syndrome with adalimumab: case report and a review of the literature. Am J Clin Dermatol, 2012. 13(1): p. 55-9.

[15]. Castellví, I., et al., Successful treatment of SAPHO syndrome with adalimumab: a case report. Clin Rheumatol, 2010. 29(10): p. 1205-7.

[16]. Sáez-Martín, L.C., et al., Etanercept in the treatment of SAPHO syndrome. Int J Dermatol, 2015. 54(6): p. e206-8.

[17]. Zhang, L.L., J.X. Zhao and X.Y. Liu, Successful treatment of SAPHO syndrome with severe spinal disorder using entercept: a case study. Rheumatol Int, 2012. 32(7): p. 1963-5.

[18]. Wagner, A.D., et al., Sustained response to tumor necrosis factor alpha-blocking agents in two patients with SAPHO syndrome. Arthritis Rheum, 2002. 46(7): p. 1965-8.

[19]. Sato, H., et al., Adult-onset Chronic Recurrent Multifocal Osteomyelitis with High Intensity of Muscles Detected by Magnetic Resonance Imaging, Successfully Controlled with Tocilizumab. Intern Med, 2017. 56(17): p. 2353-2360.

[20]. Fujita, S., et al., Development of aseptic subcutaneous abscess after tocilizumab therapy in a patient with SAPHO syndrome complicated by amyloid A amyloidosis. Int J Rheum Dis, 2015. 18(4): p. 476-9.

[21]. Sun, X.C., et al., Failure of tocilizumab in treating two patients with refractory SAPHO syndrome: a case report. J Int Med Res, 2018. 46(12): p. 5309-5315.

[22]. Ji, Q., et al., Exceptional response of skin symptoms to secukinumab treatment in a patient with SAPHO syndrome: Case report and literature review. Medicine (Baltimore), 2022. 101(33): p. e30065.

[23]. Wang, L., B. Sun and C. Li, Clinical and Radiological Remission of Osteoarticular and Cutaneous Lesions in SAPHO Patients Treated With Secukinumab: A Case Series. J Rheumatol, 2021. 48(6): p. 953-955.

[24]. Funabiki, M., et al., SAPHO Syndrome Complicated by Lesions of the Central Nervous System Successfully Treated with Brodalumab. Case Rep Rheumatol, 2023. 2023: p. 6005531.

[25]. Leloup, P., et al., Ustekinumab therapy for severe interstitial granulomatous dermatitis with arthritis. JAMA Dermatol, 2013. 149(5): p. 626-7.

[26]. Flora, A., et al., Rapid and sustained remission of synovitis, acne, pustulosis, hyperostosis, and osteitis (SAPHO) syndrome with IL-23p19 antagonist (risankizumab). JAAD Case Rep, 2021. 14: p. 33-36.

[27]. Licata, G., et al., SAPHO syndrome successful treated with tildrakizumab. Dermatol Ther, 2021. 34(1): p. e14758.

[28]. Wendling, D., C. Prati and F. Aubin, Anakinra treatment of SAPHO syndrome: short-term results of an open study. Ann Rheum Dis, 2012. 71(6): p. 1098-100.

[29]. Eleftheriou, D., et al., Biologic therapy in refractory chronic non-bacterial osteomyelitis of childhood. Rheumatology (Oxford), 2010. 49(8): p. 1505-12.

[30]. Yang, Q., et al., Case report: successful treatment of refractory SAPHO syndrome with the JAK inhibitor tofacitinib. Medicine (Baltimore), 2018. 97(25): p. e11149.

[31]. Cao, F., et al., SAPHO syndrome complicated with relapsing polychondritis: A case report. Int J Rheum Dis, 2023. 26(10): p. 2060-2063.

[32]. Yuan, F., J. Luo and Q. Yang, SAPHO Syndrome Complicated by Ankylosing Spondylitis Successfully Treated With Tofacitinib: A Case Report. Front Immunol, 2022. 13: p. 911922.

[33]. Ru, C., et al., SAPHO syndrome with Takayasu arteritis successfully treated with tofacitinib. Int J Rheum Dis, 2023. 26(7): p. 1381-1383.

[34]. Liu, Y., et al., Synovitis, acne, pustulosis, hyperostosis and osteitis syndrome with mandibular involvement: Would surgical operation help? Int J Rheum Dis, 2023. 26(3): p. 563-567.

[35]. Wang, R., et al., Synovitis, Acne, Pustulosis, Hyperostosis and Osteitis (SAPHO) Syndrome with Henoch-Schönlein Purpura: A Case Report. Clin Cosmet Investig Dermatol, 2023. 16: p. 1089-1094.

[36]. Baisya, R., et al., A Case of SAPHO Syndrome Complicated by Uveitis with Good Response to Both TNF Inhibitor and JAKinib. Case Rep Rheumatol, 2023. 2023: p. 6201887.

[37]. Yang, J., et al., Successful Treatment of Refractory Synovitis, Acne, Pustulosis, Hyperostosis, and Osteitis (SAPHO) Syndrome with Baricitinib, a Janus Kinase Inhibitor. Clin Cosmet Investig Dermatol, 2024. 17: p. 529-537.

[38]. Liu, S., et al., Efficacy of baricitinib in synovitis, acne, pustulosis, hyperostosis, and osteitis syndrome: A case series. Joint Bone Spine, 2023. 90(5): p. 105587.

[39]. Ma, M., et al., Novel JAK-1 inhibitor upadacitinib as a possible treatment for refractory SAPHO syndrome: A case report. Int J Rheum Dis, 2023. 26(11): p. 2335-2337.

[40]. Adamo, S., et al., Successful treatment of SAPHO syndrome with apremilast. Br J Dermatol, 2018. 179(4): p. 959-962.

[41]. Li, C., et al., Efficacy of bisphosphonates in patients with synovitis, acne, pustulosis, hyperostosis, and osteitis syndrome: a prospective open study. Clin Exp Rheumatol, 2019. 37(4): p. 663-669.

[42]. Amital, H., et al., SAPHO syndrome treated with pamidronate: an open-label study of 10 patients. Rheumatology (Oxford), 2004. 43(5): p. 658-61.

[43]. Kerrison, C., et al., Pamidronate in the treatment of childhood SAPHO syndrome. Rheumatology (Oxford), 2004. 43(10): p. 1246-51.

[44]. Valls-Roc, M., et al., SAPHO syndrome and pamidronate revisited. Rheumatology (Oxford), 2005. 44(1): p. 137; author reply 137-8.

[45]. Solau-Gervais, E., et al., The usefulness of bone remodelling markers in predicting the efficacy of pamidronate treatment in SAPHO syndrome. Rheumatology (Oxford), 2006. 45(3): p. 339-42.

[46]. Colina, M., R. La Corte and F. Trotta, Sustained remission of SAPHO syndrome with pamidronate: a follow-up of fourteen cases and a review of the literature. Clin Exp Rheumatol, 2009. 27(1): p. 112-5.

[47]. Delattre, E., et al., SAPHO syndrome treatment with intravenous pamidronate. Retrospective study of 22 patients. Joint Bone Spine, 2014. 81(5): p. 456-8.

[48]. Courtney, P.A., et al., Treatment of SAPHO with pamidronate. Rheumatology (Oxford), 2002. 41(10): p. 1196-8.

[49]. Marshall, H., et al., Pamidronate: a novel treatment for the SAPHO syndrome? Rheumatology (Oxford), 2002. 41(2): p. 231-3.

[50]. Guignard, S., et al., Pamidronate treatment in SAPHO syndrome. Joint Bone Spine, 2002. 69(4): p. 392-6.

[51]. Wu, N., et al., A single cohort, open-label study of the efficacy of pamidronate for palmoplantar pustulosis in synovitis, acne, pustulosis, hyperostosis and osteitis (SAPHO) syndrome. Clin Exp Rheumatol, 2020. 38(6): p. 1263-1264.

[52]. Ichikawa, J., et al., Successful treatment of SAPHO syndrome with an oral bisphosphonate. Rheumatol Int, 2009. 29(6): p. 713-5.

[53]. Liu, S., et al., Short-term efficacy of zoledronic acid in the treatment of 30 cases of SAPHO syndrome. Clin Exp Rheumatol, 2024. 42(1): p. 205-206.

[54]. Kopterides, P., D. Pikazis and C. Koufos, Successful treatment of SAPHO syndrome with zoledronic acid. Arthritis Rheum, 2004. 50(9): p. 2970-3.

[55]. Just, A., et al., Successful treatment of primary chronic osteomyelitis in SAPHO syndrome with bisphosphonates. J Dtsch Dermatol Ges, 2008. 6(8): p. 657-60.

[56]. Soyfoo, M.S., V. Gangji and J. Margaux, Successful treatment of SAPHO syndrome with ibandronate. J Clin Rheumatol, 2010. 16(5): p. 253.
